# Soy and Health Update: Evaluation of the Clinical and Epidemiologic Literature

**DOI:** 10.3390/nu8120754

**Published:** 2016-11-24

**Authors:** Mark Messina

**Affiliations:** Nutrition Matters, Inc., 26 Spadina Parkway, Pittsfield, MA 01201, USA; markjohnmessina@gmail.com; Tel.: +1-413-464-0565

**Keywords:** soy, soyfoods, nutrient content, isoflavones, cancer, heart disease, renal function, hot flashes, depression, safety

## Abstract

Soyfoods have long been recognized as sources of high-quality protein and healthful fat, but over the past 25 years these foods have been rigorously investigated for their role in chronic disease prevention and treatment. There is evidence, for example, that they reduce risk of coronary heart disease and breast and prostate cancer. In addition, soy alleviates hot flashes and may favorably affect renal function, alleviate depressive symptoms and improve skin health. Much of the focus on soyfoods is because they are uniquely-rich sources of isoflavones. Isoflavones are classified as both phytoestrogens and selective estrogen receptor modulators. Despite the many proposed benefits, the presence of isoflavones has led to concerns that soy may exert untoward effects in some individuals. However, these concerns are based primarily on animal studies, whereas the human research supports the safety and benefits of soyfoods. In support of safety is the recent conclusion of the European Food Safety Authority that isoflavones do not adversely affect the breast, thyroid or uterus of postmenopausal women. This review covers each of the major research areas involving soy focusing primarily on the clinical and epidemiologic research. Background information on Asian soy intake, isoflavones, and nutrient content is also provided.

## 1. Introduction

The health effects of soyfoods have been rigorously investigated for more than 25 years. More than 2000 soy-related peer-reviewed articles are published annually. Much of this research has been conducted because independent of its nutrient content there is evidence that soy exerts a variety of health benefits, especially related to the prevention of chronic disease.

Given the volume of research it is understandably challenging for health professionals to interpret the soy literature, a task that is compounded by the widely divergent results from in vitro and animal studies. However, these types of studies are of doubtful relevance to understanding the effects of soyfoods in humans. Obviously, in vitro conditions do not duplicate the complexity of living organisms, human or otherwise. Also, by necessity, these studies typically examine the effects of isolated compounds, which may be quite different from the effects seen when these compounds are examined in their natural milieu. Furthermore, the biological impact of one food constituent can be affected by the presence of others [1,2]. Studies in mice and rats often have limited value for predicting effects in humans because of the many physiological and anatomical differences between rodents and humans. In the case of soy, there is an additional caveat; most animals, including rodents and non-human primates, metabolize isoflavones very differently than humans [3,4,5,6,7,8,9].

Therefore, the intent of this review is to provide a synopsis of the current understanding of the health effects of soyfoods in the major areas under investigation based primarily upon the clinical and epidemiologic research. Background information on nutrient content and isoflavones, a class of compounds in soybeans that has been widely investigated, is also provided.

## 2. Nutrient Content

The macronutrient composition of the soybean differs markedly from other legumes as it is much higher in fat, moderately higher in protein and much lower in carbohydrate (Table 1) [10,11].

### 2.1 Protein

The soybean is notable not only for its total protein content but the quality of soy protein which is higher than that of other plant proteins and similar to animal protein [12]. The protein digestibility corrected amino acid scores (PDCAAS) for soy protein range from 0.9 to 1.0 depending upon the specific soyfood in question [12,13]. Although most work in this area has involved the modern soy products such as isolated soy protein (ISP is by definition at least 90% protein by weight) and soy protein concentrate, evidence indicates that the digestibility of protein from traditional Asian soyfoods is quite good [14,15,16,17,18,19]. Small differences in PDCAASs for soy protein from different soy products exist because of minor differences in amino acid profile and digestibility that result from processing and because of the presence or absence of components such as fiber and phytate [20].

Recently, the Food and Agriculture Organization of the United Nations (FAO) recommended gradually moving from the PDCAAS to another method for determining protein quality, the DIAAS, the digestible indispensable amino acid score [21]. The DIAAS value for soy protein will likely be somewhat lower than the PDCAAS value, as will be true for most proteins because the ileal digestibility of individual amino acids will be used as the measure of digestibility rather than fecal digestibility of the protein overall, but soy protein will still have a DIAAS of approximately 0.9 [13]. This value is well above the value of 0.75 which is the minimum value the FAO recommends should be allowed for nutrient claims to be made for high-quality proteins [21]. Despite recognition of the DIAAS over the PDCAAS, for a number of reasons, but in particular because of the lack of human data, it will likely be years before the former becomes accepted as the single assay of choice by regulatory bodies [22].

Finally, nitrogen conversion factors (NCFs) to determine protein content from Kjeldahl nitrogen measurements have been used for over 100 years [23]. For soy and most other proteins, this factor has traditionally been 6.25. This figure was derived from estimates of the nitrogen content of protein (16%, resulting in 100/16 = 6.25) using a limited number of proteins, but has served as an effective proxy for determining protein concentration for many proteins.

However, in recent years some have suggested that for soy protein a NCF of 5.71 should be used rather than 6.25 [24]. The 5.71 factor that is being proposed originally came from the work of D.B. Jones who published a USDA circular in 1931 on protein conversion factors. He argued that a factor of 6.25 was not appropriate for all proteins and that unique factors should be used based upon the amino acid composition of specific proteins [25]. Jones derived the 5.71 factor for soy protein based on the nitrogen content of one storage protein in soy, glycinin. However, glycinin is not representative of all proteins in soy and it has a relatively high nitrogen content [26]. Morr (1981) estimated that the NCF for different soy proteins using the Factor Method ranged from 6.70 to 6.84 [27].

The use of a NCF of 6.25 for soy is supported by an international consensus of Codex Standards, National and Regional Government Nutrition Labeling Regulations, and globally recognized standards development organizations, including the Association of Official Agricultural Chemists (AOAC) [28], the American Oil Chemist’s Society (AOCS) [29], the American Association of Cereal Chemists International (AACCI) [30] and the International Organization for Standardization (ISO) [31]. Also in the EU legislation 6.25 is the conversion factor used for protein labeling (cfr annex I Regulation 1169/2011).

### 2.2. Carbohydrate

The low carbohydrate content of the soybean means that many traditional Asian soyfoods are also low in this macronutrient which may make them advantageous for people with diabetes [32].

Also, much of the soybean carbohydrate is comprised of oligosaccharides (predominately stachyose) [33,34,35,36], which, because they are poorly digested by intestinal enzymes, travel to the colon where they are able to stimulate the growth of bacteria such as bifidobacteria, that are considered to be advantageous to the host. For this reason the soybean oligosaccharides are classified as prebiotics [37,38,39]. Twenty-five years ago, a soybean oligosaccharide extract was proposed as being a healthy alternative to table sugar [40].

Although oligosaccharides can lead to flatulence [41], research suggests that concerns about the flatulence-producing effects of beans may be exaggerated [42]. Furthermore, this concern is not applicable to many soy products because as a result of processing the oligosaccharide content is greatly reduced or eliminated. This is true for example for tempeh [43], tofu [43] and ISP [41] but not soy grits and soy protein concentrate [41].

### 2.3. Fat

The fat content of soybeans is comprised of approximately 10%–15% saturated, 19%–41% monounsaturated and 46% to 62% polyunsaturated fatty acids (PUFA) (per 100 g soybeans that equates to approximately 2.9, 4.4 and 11.3 g saturated, monounsaturated and PUFA, respectively) (Table 2) [44]. As can be seen from Table 2 these figures vary considerably within the same variety and among different varieties. The PUFA is comprised of the omega-6 essential fatty acid linoleic acid and α-linolenic acid, the essential omega-3 fatty acid. The ratio of these two fatty acids is approximately 7–8:1 but the range is from approximately 5–13:1 (Table 2). The soybean and full-fat soyfoods are some of the few foods that are good sources of both essential fatty acids. Because of its widespread use in the United States, soybean oil accounts for over 40% of the intake of both essential fatty acids [45].

### 2.4. Vitamins and Minerals

The soybean is a good source of a variety of vitamins and minerals, especially for example, potassium (Table 1) [10,11]. The potassium content is notable because intake of this mineral is often suboptimal [46]. It is important to recognize however that the nutrient content of individual soy products, depending upon the manufacturing method employed can differ markedly from one another and from the bean itself. For example, because the traditional Japanese soyfood natto is produced by fermentation using a strain of *Bacillus subtilis*, it is an extremely rich source of vitamin K_2_ [47,48].

Soybeans are relatively high in iron (Table 1) which is not surprising because although it is often not recognized, the iron content of plant-based diets is similar to [49] or higher [50] than the iron content of omnivorous diets. Issues surrounding mineral status of those consuming plant-based diets often have more to do with absorption efficiency than intake. The absorption of calcium and iron from soy is an important consideration because soyfoods often replace foods that are good sources of these minerals. Surprisingly, despite the presence of both phytate and oxalate, two compounds that inhibit mineral absorption, calcium absorption from soybeans is quite good [51]. For example, Heaney et al. (1991) found that the fractional calcium absorption from cow’s milk was 0.377 compared with 0.310 and 0.414 for high- and low-phytate soybeans, respectively [51]. Zhao et al. (2005) reported that the fractional calcium absorption from cow’s milk (0.217) did not differ from calcium carbonate-fortified soymilk (0.211) although it was slightly higher than from tricalcium phosphate-fortified soymilk (0.181) [52]. In agreement, Tang et al. (2010) found calcium absorption from soymilk [53] and Weaver et al. (2002) found calcium absorption from tofu made by coagulating soymilk with a calcium salt such as calcium chloride or calcium sulfate was similar to the absorption of calcium from cow’s milk [54].

The traditional view that the iron in soyfoods and in essentially all plant foods is poorly absorbed is contradicted by relatively new research utilizing improved methodology that shows iron absorption from soy may be quite good because most of it is in the form of ferritin [55,56]. Furthermore, in contrast to older understanding [57], there appears to be adaptation to the inhibitory effects of phytate on iron absorption [58]. Therefore, acute absorption studies may underestimate the bioavailability of minerals from plant foods such as soy. It should also be noted that iron status greatly influences nonheme iron absorption with higher absorption rates being observed in individuals with low iron stores [59].

## 3. Isoflavones

Isoflavones are found in many different plant foods but among commonly consumed foods the soybean contains uniquely rich amounts. This point that is illustrated by the difference in isoflavone intake between Asian and non-Asian countries. For example, mean isoflavone intake among adults ranges from about 30–50 mg/day in Japan but is less than 3 mg/day in the United States, Canada and Europe [60,61,62,63,64,65,66,67].

Isoflavones occur in soybeans almost exclusively as glycosides [68] but upon ingestion, the sugar is hydrolyzed thereby allowing absorption to occur [69]. In fermented soyfoods such as miso, tempeh and natto, considerable amounts of the isoflavones, although to varying degrees occur as aglycones due to bacterial hydrolysis [70,71,72,73]. The three isoflavones genistein, daidzein and glycitein and their respective glycosides account for approximately 50%, 40% and 10%, respectively, of the total isoflavone content of soybeans [68]. There is disagreement over whether isoflavone form affects total absorption; some studies show that in comparison to the glycoside, aglycone isoflavones are absorbed faster and to a greater extent [74,75,76] whereas other studies show aglycones are absorbed more quickly but total absorption is the same or even less [77,78,79]. In this document isoflavone amounts are expressed in aglycone equivalent weights.

In regard to isoflavone metabolism, a striking difference among individuals is that only about 25% of non-Asians and 50% of Asians host the intestinal bacteria that convert daidzein into the isoflavonoid equol [80]. In 2002, Setchell et al. (2002) proposed that those individuals who host these bacteria are more likely to benefit from soyfood consumption [81]. Since that time this hypothesis has been hotly debated. Equol does appear to offer health benefits over its precursor daidzein [82,83] in possibly several different areas but especially in the alleviation of hot flashes [84,85,86]. However, it also may be that in some cases it is the equol-producing phenotype (i.e., the ability to produce equol) rather than equol itself, that is responsible for the more beneficial response to isoflavone ingestion [87]. Rodents and non-human primates have been referred to as equol machines because of their ability to so efficiently produce such large amounts of equol in response to daidzein exposure [9]. Several excellent reviews of equol have recently been published [80,82,86,88].

Each gram of soy protein in soybeans and traditional soyfoods is associated with approximately 3.5 mg of isoflavones [89]. Consequently, one serving of a traditional soyfood, such as 100 g of tofu or 250 mL soymilk, typically provides about 25 mg isoflavones. In more refined products, such as ISP, as much as 80% to 90% of the isoflavone content can be lost as a result of processing [68,90,91].

Isoflavones have a chemical structure similar to the hormone estrogen which allows them to bind to both estrogen receptors (ER)—ERα and ERβ [92,93]. For this reason they are able to exert estrogen-like effects under certain experimental conditions and so are referred to as phytoestrogens. Note that although research shows isoflavones bind to ERs much more weakly than estrogen, circulating levels of isoflavones in response to the ingestion of approximately two servings of soyfoods are three orders of magnitude higher than estrogen [94]. More importantly, whereas estrogen binds to and transactivates ERα and ERβ equally, isoflavones preferentially bind to and transactivate ERβ [95,96,97,98]. This difference in binding and transactivation between isoflavones and estrogen is significant because the two ERs have different tissue distributions and, when activated, can have different and sometimes even opposite physiological effects [99,100].

The preference of isoflavones for ERβ is the primary reason that isoflavones are seen as capable of having tissue-selective effects and the reason they are classified as selective estrogen receptor modulators (SERMs) [101,102,103]. In tissues that possess estrogen receptors, SERMs exert estrogen-like effects in some cases but no effects or antiestrogenic effects in others. The pharmaceutical industry has for many years been actively developing SERMs [104]. Widely used SERMs include tamoxifen and raloxifene, both of which are used to treat breast cancer, the latter of which is also used for the treatment of osteoporosis [105]. There is limited clinical evidence to suggest that dietary levels of isoflavones affect immune function although there is interesting in vitro and animal work on this topic [106]. It is notable in this regard that Ryan-Borchers et al. (2006) found an isoflavone intervention (~70 mg/day) in postmenopausal women resulted in higher B cell populations which they interpreted as a heightened humoral response [107].

Although isoflavones are purported to exert a number of health benefits these molecules are not without controversy. Concerns have arisen that because of their estrogen-like properties they may exert untoward effects in some individuals such as postmenopausal women. However, after a comprehensive, multi-year evaluation of the literature, the European Food Safety Authority (EFSA) concluded that in postmenopausal women, isoflavones do not adversely affect the three organs that were investigated, the breast, thyroid and uterus [108]. Recently, the North American Menopause Society also concluded that isoflavones do not increase risk of breast or endometrial cancer.

In any event, it is clear that isoflavones should not be equated with the hormone estrogen. The literature is replete with clinical examples of differences between these two molecules. For example, isoflavones do not stimulate the vaginal maturation index [109] or increase C-reactive protein (CRP) [103] whereas estrogen does [103,109]. Furthermore, isoflavones may exert potentially-relevant hormone-independent physiological effects [110]. Therefore, classification only related to their hormonal activity may be an incomplete characterization [111]. Finally, not only should isoflavones not be equated with estrogen but soyfoods should not be equated with isoflavones. The soybean, like all foods, is a collection of many biologically active molecules [112].

## 4. Effects of Soy Protein on Cardiovascular Disease Risk Factors

### 4.1. Lipid Levels

Elevated low-density lipoprotein (LDL)-cholesterol is a well-established risk factor for coronary heart disease (CHD) [113]. As recently articulated by Jarcho and Keaney (2015), data continue to support LDL-cholesterol lowering as a primary strategy to prevent CHD [114].

Clinical research demonstrating the hypocholesterolemic effects of soy protein dates back to 1967 [115]. This benefit of soy protein was first formally recognized by the US Food and Drug Administration (FDA) in 1999 [116]. The FDA established 25 g/day soy protein as the threshold intake for cholesterol reduction. Twenty-five g is certainly more soy protein than is consumed by most Asians but through a combination of soyfoods this threshold can be relatively easily met. Since the FDA claim was approved, more than 10 countries [117] have approved similar claims including Canada, which did so in 2014 [118]. Nevertheless, there exists some controversy about the hypocholesterolemic effect of soy protein.

In general, about 20% of individuals whose cholesterol levels are elevated do not respond to dietary changes [119]. So it is not surprising, especially when considering that many interventions involved small participant numbers and that the effect of soy protein is modest, that not all studies reported soy protein statistically significantly lowered circulating cholesterol levels. In addition, since most studies have intervened with ISP less is known about the hypocholesterolemic effects of soy protein contained in other soy products such as soy flour [120]. The roots of the controversy can in many respects be traced back to 2006, when the American Heart Association (AHA) concluded that soy protein only lowers LDL-cholesterol three percent [121]. One year later, the FDA announced their intention to reevaluate the soy protein health claim.

Often overlooked is that the AHA did not actually conduct a formal statistical analysis of the data. When this was done, Jenkins et al. (2010) found using the 22 studies upon which the AHA based their conclusion, that the AHA had underestimated the cholesterol-lowering effect of soy protein [122]. They determined that soy protein lowered LDL-cholesterol 4.3% (5.2% among the high-quality studies) [122], which is in line with the results of numerous meta-analyses published over the past decade or so, that have found soy protein statistically significantly lowers LDL-cholesterol by approximately 4% to 6% (Table 3) [118,122,123,124,125,126,127,128,129,130]. Evidence indicates that the decrease in LDL-cholesterol in response to soy protein is greater in hypercholesterolemic compared to normocholesterolemic individuals [123,125,126].

In addition to lowering LDL-cholesterol, soy protein also modestly lowers circulating triglyceride levels (~5%) and raises high-density-lipoprotein (HDL)-cholesterol levels (~1%–3%) (Table 3) [123,125].

Although the lipid effects of soy protein are much more modest than initially reported [131] they are still relevant at the clinical and population levels as epidemiologic and intervention data suggest that for every 1% reduction in LDL-cholesterol there is a corresponding 1%–2% reduction in cardiovascular events (CVEs), and for every 2%–3% increase in HDL-cholesterol there is a reduction in CVEs by 2%–4% that is independent of LDL-cholesterol [132,133,134]. It should be noted that despite inverse associations between HDL-cholesterol and CVD risk noted in epidemiologic studies, recent evidence strongly calls into question the value of raising levels of this lipoprotein as means of protection against heart disease [135,136]. The mechanism(s) by which LDL-cholesterol is lowered has not been definitively identified although some work suggests peptides formed from the digestion of soy protein upregulate hepatic LDL receptors [137,138]. Finally, as discussed below, in addition to the direct effect of the protein, soyfoods potentially reduce cholesterol via substitution or replacement effects [122].

### 4.2. Blood Pressure

Higher-protein diets in general appear to modestly lower blood pressure [139] and there are clinical data suggesting soy protein in particular is hypotensive. The public health benefits of even the modest proposed hypotensive effects of soy protein are relevant as reducing systolic blood pressure by just 2–5 mmHg may reduce stroke and CHD disease by 6%–14% and 5%–9%, respectively [140].

Two trials noted extremely robust reductions in response to soy; one compared the effects of 25 g/day soy protein from soynuts with a diet containing a similar amount of protein in postmenopausal women [141] and the other compared the effects of one liter per day of soymilk with one liter of cow’s milk in men [142]. However, in the vast majority of trials the reductions reported are much more modest. Meta-analyses show systolic and diastolic blood pressure is reduced by approximately 2.5 and 1.5 mmHg, respectively [128,143,144,145]. An important caveat about these data is that in most studies blood pressure was not the primary endpoint of interest. Therefore, additional research is needed before definitive conclusions can be made. The mechanism(s) by which soy protein exerts its hypotensive effects has not been identified.

### 4.3. Endothelial Function (Vasodilation)

The endothelium is the monolayer of endothelial cells lining the lumen of the vascular beds and is mechanically and metabolically strategically located, separating the vascular wall from the circulation and the blood components. Evidence suggests that endothelial dysfunction is associated with CVEs [146].

Two meta-analyses, one published in 2011 [147] and the other in 2012 [148] found that soybean isoflavones improved endothelial function in postmenopausal women. When the data from one of these were sub-analyzed, the improvement was only found in those women who had impaired endothelial function at baseline [147]. Of course, these women are at greater risk of having or developing CHD. This finding provides at least a partial explanation for the inconsistent literature in that some studies included women with impaired endothelial function and others with normal function. Parenthetically, it may also be that some of the observed anti-inflammatory effects of isoflavones occur only in people at risk of CHD who have elevated levels of inflammatory markers [149]. Interesting, Hoshida et al. (2011) found that isoflavones (50 mg/day) improved endothelial function in pre- and postmenopausal female non-smokers but not smokers [150].

### 4.4. Arterial Stiffness

Unlike endothelial-mediated vasodilation (primarily nitric oxide-dependent), arterial stiffness relates to the constriction and dilation of arteries associated with systole and diastole. Arterial stiffness is determined by components of the artery wall, such as elastin, proteoglycans and smooth muscle cell function. The most straightforward, valid, and reliable measure of arterial stiffness is pulse wave velocity, which is predictive of future CVEs [151].

In 2011, a systematic review by Pase et al. [152] concluded on the basis of five studies [153,154,155,156,157] that isoflavones reduce arterial stiffness although one of the four that reported benefit intervened with the isoflavone metabolite equol [157]. Three studies not reviewed by Pase et al. [152] are also supportive of the ability of isoflavones to improve arterial stiffness in postmenopausal women [158,159,160]. Conversely, no differences in arterial stiffness were noted in a small trial of hypercholesterolemic men and women when soymilk/soy yogurt was compared with dairy milk/yogurt [161].

### 4.5. C-Reactive Protein

C-reactive protein, one important indicator of inflammation, is considered to be a risk marker and predictor of CVD [162,163]. A meta-analysis of 14 intervention trials showed a slight, but not significant, reduction of 0.17 mg/L (*p* = 0.12) in CRP concentrations among postmenopausal women with soy isoflavone intervention compared with controls. Subgroup analyses showed that isoflavones significantly lowered CRP by 0.70 mg/L (*p* = 0.003) among women with baseline CRP concentrations greater than 2.2 mg/L, which was equivalent to an approximate 22% reduction in levels of this inflammatory marker, whereas no change was noted in women with lower baseline CRP levels [164]. Subsequently published research also reported decreases in CRP in response to various soy interventions (soy protein, soyfoods or isoflavones) including studies involving participants engaged in an exercise program [165], subclinical hypothyroid patients [166], diabetic patients [167,168], patients with liver disease [169] and patients with renal disease [169,170]. Conversely however, soy was without effect in several other studies [120,160,171,172,173,174,175,176].

Given the inconsistency of the data, the different intervention products and experimental designs employed and that in most studies CRP was not the primary outcome of interest, it is not possible to make any definitive conclusions about the impact of different soy products on CRP. A more detailed examination of the data aimed at identifying explanations for the inconsistency is warranted.

### 4.6. Carotid Intima Media Thickness

Subclinical atherosclerosis can be assessed using ultrasound to measure the thickness of the carotid arteries. Carotid intima-media thickness (CIMT) increases over time; the extent of progression reflects risk of future coronary events. In 2011, a secondary analysis of a large 3-year trial by Hodis et al. [177] involving 350 healthy postmenopausal women (45–92) found that CIMT progression was inhibited in those consuming 25 g/day soy protein (91 mg isoflavones) compared to those women consuming 25 g/day milk protein. However, this benefit was observed primarily only in young postmenopausal women (<5 years since menopause) which is consistent with the “estrogen window” or “estrogen timing” hypothesis [178]. In contrast, a 6-month study involving Chinese postmenopausal women saw no effect of soy flour (40 g/day soy protein, ~50 mg isoflavones) on CIMT [179]. Whether the failure to observe a reduction in CIMT is because of the short study duration, lower dose of isoflavones or because the data were not subanalyzed according to years since menopause is unclear.

## 5. Effects on Cardiovascular Events

CHD and stroke account for over 20% of deaths worldwide but there are striking variations in age-adjusted cardiovascular disease (CVD) mortality rates among countries [180]. These international variations are not due primarily to genetic differences among populations as evidenced from time trends in rates within countries and changes in rates among migrants moving from low-risk to high-risk countries [181].

There has been limited epidemiologic investigation of the relationship between soy intake and CVD. A recently published meta-analysis by Lou et al. (2016) identified five cohort (3 Asian, 2 non-Asian) and three case-control studies that examined the association between soy and CHD [182]. These authors found that when comparing high vs. low intake categories, the summary relative risk (95% confidence intervals, CI) for the three case-control studies [183,184,185] was 0.66 (0.56–0.77).

With respect to the Asian cohort studies, after controlling for numerous potentially confounding variables and when comparing the extremes of intake, two found that among women soy intake was associated with marked reductions in CHD risk. One of these is from China (relative risk [RR]) plus 95% CI; 0.25: 0.10, 0.63) [186] and the other is from Japan (RR, 0.55; 95% CI: 0.26, 1.09) [187]. However, a large Singaporean prospective study failed to find soy intake was protective [188]. The aforementioned meta-analysis found the summary RR (95% CI) for these three studies was 0.57 (0.27–1.19). In contrast to the effects in women, none of the three cohort studies [187,188,189] involving Asian men found soy intake was associated with a reduced risk of CHD although one of these was published only as a letter to the editor [189].

Lou et al. (2016) identified three cohort [65,187,188] and four case-control [185,190,191,192] studies that examined the relationship between soy intake and stroke risk [182]. The summary RR (95% CI) for the case control studies, all of which involved Asian participants, was 0.54 (0.34, 0.87). However, the summary RR (95% CI) for the two of three cohort studies that involved Asian participants was 0.89 (0.71–1.12).

Soyfoods potentially reduce risk of CVD through multiple mechanisms. It is noteworthy that in a recent review, Nabavi et al. (2015) concluded that genistein decreases the chance of ischemic stroke through reduction in ischemic stroke risk factors and ameliorates ischemic stroke induced neuronal damages [193]. Also, as noted previously, soy protein lowers LDL-cholesterol [118,123,124,125] and possibly blood pressure and isoflavones may improve arterial health [128,143,144,145].

In addition, when soyfoods replace protein-rich foods that are sources of saturated fat, which will likely be the case in Western cultures, because of the change in the fatty acid profile of the diet, LDL-cholesterol and CHD risk will likely be reduced. One estimate, based upon the US National Health and Nutrition Examination Survey III population data, is that when 24 g soy protein from soyfoods replace a similar amount of non-soy protein, through displacement effects LDL-cholesterol will be reduced approximately 4% (similar to the direct effect of soy protein) [122].

Although there has been recent controversy about the relationship between dietary fat and CHD [194,195] health agencies and organizations continue to recommend limiting saturated fat intake [196,197]. Recent support for this recommendation comes from a combined analysis of the Nurses' Health Study (study years, 1980 to 2010; *n* = 84,628) and the Health Professionals Follow-up Study (study years, 1986–2010; *n* = 42,908 men) which found that replacing 5% of energy intake from saturated fat with equivalent energy intake from PUFA, monounsaturated fat, or carbohydrates from whole grains was associated with a 25%, 15%, and 9% lower risk of CHD, respectively, whereas replacing saturated fat with carbohydrates from refined starches/added sugars was not protective [198].

Claims that the high omega-6 content of soybean oil and other plant oils produce a pro-inflammatory effect are not supported by the clinical evidence [199,200,201,202]. One recently published often-cited but controversial analysis concluded that only trials intervening with oils containing a mix of linoleic acid and omega-3 PUFAs reduce risk [203]. If this observation proves to be correct, soybean oil and full-fat soyfoods will be considered heart-healthy as a result of their fatty acid profile because as previously highlighted, the soybean is one of few foods that provides ample amounts of both omega-6 and omega-3 fatty acids.

The extent to which the hypocholesterolemic and hypotensive effects of soy protein contribute to the reductions in CHD noted in several of the observational studies is not clear. It is notable that the protective effects observed in the previously cited Japanese [187] and Chinese [186] cohort studies were greater than could be expected based on the mean reductions in blood pressure and LDL-cholesterol in response to soy protein observed in clinical trials. Furthermore, even in the upper categories soy protein intake was less than the amounts thought necessary to affect these endpoints.

Since CVD protection was observed in women and not men, it is certainly possible that the isoflavones in soyfoods played an important role in this regard. As previously discussed, in postmenopausal women, these soybean constituents have been shown to improve arterial stiffness [152] and flow mediated dilation [147]. In epidemiologic studies showing soy is protective against CVD, estimated isoflavone exposure is not dissimilar from the doses used in clinical studies which show benefits on arterial health.

## 6. Bone Health

In response to declining estrogen levels, women can lose substantial amounts of bone mass in the decade following menopause, which markedly increases their fracture risk [204]. Estrogen therapy reduces postmenopausal bone loss and hip fracture risk by approximately one-third [205]. Initial speculation that soyfoods promote bone health in postmenopausal women was based on the estrogen-like effects of isoflavones and early research showing that the synthetic isoflavone, ipriflavone, exerted skeletal benefits [206].

Two large Asian prospective epidemiologic studies found among women soy intake was associated with a one-third reduction in fracture risk [207,208]. A third prospective epidemiologic study, which involved US Seventh-day Adventists (approximately 40% of whom are vegetarians), found that among postmenopausal women soymilk consumption was associated with a significantly lower risk of developing osteoporosis but since the intake of cow’s milk was similarly protective, the benefit of soy may have been due to its calcium rather than isoflavone content [209].

Many short-term clinical studies have found that isoflavones favorably affect bone turnover and/or bone mineral density (BMD) in postmenopausal women [210,211,212]. In contrast, of the four large, long-term clinical trials that have been conducted [213,214,215,216], only one trial, which involved osteopenic women, found isoflavones statistically significantly improved BMD [213]. However, a recently published clinical study which employed novel methodology to assess bone calcium content not only supports the efficacy of isoflavones but provides a possible explanation for the disappointing results from the long-term trials [217].

In this cross-over study, over a 50-day period, isoflavones (105 mg/day) increased bone calcium content by 7.6% (*p* = 0.0001), approximately half the increase noted in response to risedronate, a bisphosphonate used in the treatment of osteoporosis [218]. Furthermore, doubling the dose of isoflavones to amounts similar to those used in two of the long-term trials [215,216], actually increased bone calcium content to a much lesser extent than the lower dose [217]. Therefore, more mderate doses of isoflavones (50–100 mg/day) may prove to be more efficacious for promoting bone health than more pharmacologic doses.

Although at this point more research is required to establish that isoflavones exert skeletal benefits, the high-quality protein [12,13] and well-absorbed calcium [52,53] provided by many soyfoods certainly can contribute to bone health.

## 7. Breast Cancer

### 7.1. Risk Reduction

It is widely recognized that breast cancer incidence rates in soyfood-consuming countries are much lower than in Western countries [219]. Furthermore, as Westernization of Asian cultures has occurred, Asian breast cancer rates have steadily increased [220]. While these observations helped fuel interest in the chemopreventive effects of soy isoflavones, more relevant are Asian epidemiologic studies which show that higher soy consumption is associated with an approximate one-third reduction in breast cancer risk although most data come from case-control not longitudinal studies [221]. However, considerable data suggest that for soy to reduce risk consumption must occur during childhood and/or adolescence [222,223,224,225,226,227,228]. Case-control studies show higher soy intake early in life is associated with 25% to 60% reductions in risk [225,226,227,228].

The “early intake” hypothesis, which was first proposed in 1995 [222,223], is consistent with clinical studies showing that in adults, neither soy protein nor isoflavone supplements affect markers of breast cancer risk including mammographic density [229,230] and breast cell proliferation [231,232,233,234,235,236]. It is also consistent with the recognition of the growing evidence linking childhood and adolescent lifestyle and environmental exposures with subsequent risk of cancers arising in adulthood [237].

The recent results by Baglia et al. (2016) from the Shanghai Women’s Health Study provide interesting insight into the impact of timing of soy intake on breast cancer risk [238]. After a median follow-up period of 13.2 years, 1034 incident breast cancer cases were identified among the >70,000 women enrolled in this study. When women were divided into three soy protein intake groups (low, medium, and high) it was found that high intake during both adolescence and adulthood significantly reduced breast cancer risk (hazard ratio [HR], 0.53; 95% CI: 0.32, 0.88). However, consuming higher amounts of soy only during adolescence (and low soy intake during adulthood) was almost as protective (HR, 0.56; 95% CI: 0.31, 1.00). Thus, these results support the early intake hypothesis. Interestingly, high soy protein intake during adulthood was only protective (HR, 0.63; 95% CI: 0.43, 0.91) against breast cancer among women who consumed little soy during adolescence. Baglia et al. (2016) suggest the reason for this finding is that protective effects would have already been manifest against breast cancer among premenopausal women if they had consumed soy when young [238].

The low intake among non-Asians severely limits the ability of Western epidemiologic studies involving the general population to provide meaningful data about the health effects of soy [239]. However, results from the Oxford arm of the European Prospective Investigation into Cancer and Nutrition (EPIC) have been cited as supporting the early intake hypothesis [240]. This study oversampled for vegetarians so soy intake among a sizeable percentage of the participants was similar to that reported in Japanese studies. It has been argued that the Oxford-EPIC found no association between isoflavone intake and breast cancer risk because the high-soy-consumers in this cohort likely began eating soy only later in life with their adoption of a vegetarian diet [241].

Finally, several mechanisms for the protective effects of early isoflavone exposure have been proposed [242,243,244,245,246]. The protection afforded by isoflavones may be similar to the observed protective effect of early pregnancy against breast cancer [242].

### 7.2. Breast Cancer Patients

The estrogen-like effects of isoflavones provided a theoretical basis for concern that soyfoods adversely affect the prognosis of breast cancer patients. However, not only as discussed previously, do isoflavones differ from the hormone estrogen but the evidence that estrogen therapy increases breast cancer risk is unimpressive. In fact, in the Women’s Health Initiative trial, which involved over 10,000 women, estrogen therapy statistically significantly reduced risk of developing invasive breast cancer [247].

Nevertheless, in ovariectomized athymic mice implanted with MCF-7 cells, an estrogen-sensitive human breast cancer cell line, isoflavones stimulate the growth of existing mammary tumors [248,249]. However, because rodents metabolize isoflavones differently than humans, the value of these studies for understanding effects in humans is in doubt [3,4,5,6,7,8]. Furthermore, not all rodent models show that soy or isoflavones stimulate the growth of existing mammary tumors [250,251,252] and even in the aforementioned rodent model that does, minimally processed soy does not have this effect [253]. In addition, just slightly tweaking this model in a more physiologic direction causes a complete loss of the stimulatory effects of isoflavones [251].

More importantly, the clinical data show that soy isoflavones, regardless of the source, and even when exposure greatly exceeds Japanese intake, do not exert harmful effects on breast tissue [229,230,231,232,233,234,235,236]. These findings are consistent with the conclusion of the EFSA although their review focused on healthy postmenopausal women not breast cancer patients [108]. In addition to the lack of effect on mammographic density and breast cell proliferation, neither soy nor isoflavone supplements have clinically relevant effects on reproductive hormones in women [254].

Finally, the prospective epidemiologic data show that post-diagnosis soy intake improves prognosis. More specifically, a meta-analysis of five prospective studies, two from the United States [255,256] and three from China [257,258,259], involving over 11,000 women with breast cancer, found soy consumption after a diagnosis of their disease was associated with statistically significant reductions in breast cancer recurrence (HR, 0.85; 95% CI: 0.77, 0.93) and mortality (HR, 0.79; 95% CI: 0.72, 0.87) (Table 4) [260]. Importantly, soy consumption was similarly beneficial in Asian and non-Asian women [261]. Also, in contrast to the results in ovariectomized athymic mice showing genistein negates the inhibitory effect of tamoxifen [262,263] and an aromatase inhibitor [264] on mammary tumor growth, the prospective epidemiologic data suggest that soyfood intake may actually enhance the efficacy of these treatments [258,261].

The positions of the American Cancer Society [265] and the American Institute for Cancer Research [266] are that soyfoods can be safely consumed by women with breast cancer. In addition, an evidence-based conclusion in response to a recent clinical inquiry published in the Journal of Family Practice was that post-diagnosis soy intake improves the prognosis of breast cancer patients [267]. This conclusion is similar to that of the World Cancer Research Fund International that there is a possible link between consuming soyfoods and improved breast cancer prognosis [268].

## 8. Prostate Cancer

Prostate cancer is the second most commonly diagnosed cancer in men worldwide, and the fourth most commonly diagnosed cancer overall [269]. However, as is the case for breast cancer, prostate cancer incidence and mortality rates vary dramatically throughout the world; rates in Asian countries where soyfoods are commonly consumed are very low relative to Western countries [270]. More relevant are the Asian population studies showing that higher soy consumption is associated with as much as a 50% reduction in prostate cancer risk although most data come from case-control not longitudinal studies [271,272,273,274].

Intervention studies involving prostate cancer patients generally show that isoflavone exposure slows the rise in prostate specific antigen (PSA) levels [275,276,277,278]. In contrast, in long-term trials isoflavone exposure did not affect the biochemical recurrence of prostate cancer after radical prostatectomy [279] or the progression from high-grade prostatic intraepithelial neoplasia to cancer [280]. However, the design weaknesses of each study limit the implications of the findings. In the former, patients were exposed to only 24 mg/day genistein, a relatively low dose for men who had already undergone radical prostatectomy for the treatment of prostate cancer [279]. In the other study, in addition to the treated group consuming isoflavone-rich soy protein, participants were administered supplements of vitamin E and selenium [280]. There is evidence that both of these micronutrients stimulate the development of prostate cancer, although the risk associated with vitamin E is much less clear [281,282].

In regard to possible mechanisms, since neither soy intake nor isoflavone exposure affects testosterone levels in men, effects on this hormone are not a possible explanation for the proposed protective effects of soy against prostate cancer [283]. There is some evidence from animal and clinical studies that isoflavones are able to inhibit metastasis [284,285]. Also, it was suggested more than a decade ago that genistein exerts some of its proposed chemopreventive effects through binding to ERβ [286]. ERβ is expressed in prostate epithelial cells and has a role in cellular homeostasis that is anti-proliferative [287], pro-differentiative [288], and pro-apoptotic [289].

## 9. Kidney Function

The potential renal benefits of soy have considerable public health significance because of the increasing worldwide prevalence of renal disease, which is largely a consequence of the increasing prevalence of diabetes [290]. There is preliminary evidence that soy protein places less stress on the kidneys in comparison to other high-quality proteins, which over time could reduce the risk of developing renal disease in susceptible individuals, such as those with diabetes [291,292]. More specifically, it has been proposed that replacing animal protein with soy protein leads to a decrease in hyperfiltration and glomerular hypertension, with resultant protection from diabetic nephropathy [291,293].

However, a recent meta-analysis of 12 clinical studies involving 280 patients with chronic renal disease found that dietary soy protein did not affect glomerular filtration rate although it significantly decreased serum creatinine, serum phosphorus, inflammation (assessed by CRP) and proteinuria in predialysis patients [294]. In individuals whose renal function is compromised serum phosphorus levels often become abnormally high so replacing animal protein with soy protein could be helpful [295]. For more detailed information about the renal effects of soyfoods the reader is referred to the reference [296].

## 10. Menopausal Symptoms

Hot flashes are the most common reason given by women seeking treatment for menopausal symptoms. Although hot flashes usually subside within six months to two years [297,298], many women report having them for up to 20 years after menopause [299]. It is notable that in the Study of Women’s Health Across the Nation, a multiracial/multiethnic observational study of the menopausal transition involving 3302 women enrolled at seven US sites, among women who began having hot flashes during the pre- or perimenopausal period, the average duration of hot flashes was 11.8 years [300].

The low prevalence of hot flashes among native Japanese women combined with the knowledge that isoflavones interact with ERs led to the hypothesis that soyfoods prevent the onset of hot flashes and/or are capable of alleviating existing hot flashes [301]. The first trial to test this hypothesis was published more than two decades ago [302]. Since then at least 25 trials have evaluated the effects of a variety of soy products on menopause-related hot flashes although in recent years most trials have intervened with isoflavone supplements [303,304]. These trials have produced inconsistent results. However, a meta-analysis published in 2012 provides strong support for the efficacy of isoflavones as well as an explanation for the inconsistent data [305].

This analysis found that soybean isoflavones statistically significantly reduced the frequency and severity of hot flashes by 20.6% (*p* < 0.00001) and 26.2% (*p* = 0.001), respectively, above and beyond the reduction that occurred in the placebo group (total reduction in response to isoflavones, i.e., placebo plus isoflavones, ~50%). However, sub-analysis of the data revealed that in the trials in which the intervention supplement provided >18.8 mg genistein (the median for all trials) the reduction in hot flash frequency was more than twice that observed in those trials involving supplements providing lesser amounts of genistein (Table 5). Approximately 40 mg total isoflavones derived from whole soybeans provides the amount of genistein shown to be efficacious.

Failure to differentiate between low-genistein (typically derived from soy germ) and high-genistein (typically derived from the whole soybean) supplements, as is the case for all other narrative reviews and meta-analyses of data with one exception [306], leads to erroneous conclusions about the efficacy of isoflavones.

## 11. Cognitive Function

The impact of both cumulative estrogen exposure and estrogen therapy on cognitive function and Alzheimer’s disease (AD) is controversial although most recent work does not support the notion that estrogen affords significant benefits in this regard [307,308,309,310].

An intriguing theory proposed more than a decade ago is that exposure to estrogenic molecules soon, but not late after menopause, produces pronounced cognitive benefits [178]. Since isoflavones interact with ERs there has been interest in understanding the impact of isoflavone exposure on cognitive function. Unexpectedly, an early epidemiologic study that was designed to study heart disease, raised concern about soy adversely affecting cognition [311], a finding that was supported by more recent research from Indonesia [312]. However, follow up research from Indonesia produced opposite results [313] and epidemiologic studies overall that have evaluated the impact of soy intake on cognition have produced very mixed findings [314,315,316,317].

In contrast to the epidemiologic data, a meta-analysis of 10 placebo-controlled randomized clinical trials involving 1024 participants found that soy isoflavones favorably affected summary cognitive function and visual memory in postmenopausal women [318]. However, a 3-year trial involving over 300 postmenopausal women failed to show that isoflavone-rich soy protein affects global cognition [319]. More recently, a six-month study found that 100 mg/day isoflavones did not benefit cognition in older men and women with AD [320]. After comprehensively evaluating the animal, clinical and epidemiologic data, Soni et al. (2014) recently concluded that “the evidence to date is not sufficient to make any recommendations about the association between dietary intake of soy isoflavones and cognition in older adults” [321]. It seems reasonable to assume that at this point this conclusion will be valid for the foreseeable future.

## 12. Mental Health

There are several areas in relation to the impact of soy intake that have been investigated to only a limited extent but that based upon preliminary results warrant additional research. One such area is mental health and specifically depression. Depression is a commonly occurring disorder associated with diminished quality of life and increased morbidity and mortality [322,323]. Strikingly, there is an approximate two-fold female-male disparity in the prevalence of depression [324]. The higher prevalence of depression among women compared to men suggests that reproductive hormones may be involved in the etiology of this disease. Also, longitudinal studies suggest that menopause is a period of risk for new onset or recurrent depression for some women [325,326,327]. Importantly, there is a growing recognition that diet affects mental health; in fact, a group of academics recently concluded that “diet is as important to psychiatry as it is to cardiology, endocrinology, and gastroenterology” [328].

With this background in mind the emerging evidence suggesting that isoflavones may function as antidepressants is particularly intriguing [329]. For example, over a two-year period, an Italian study that was evaluating mood effects, found that postmenopausal women taking 54 mg/day genistein showed a decline in depressive symptoms as measured by the Zung Self-rating Depression Scale whereas no change occurred in the placebo group [330]. Also, a Japanese study involving peri- and postmenopausal women found that a very moderate dose (25 mg/day) of isoflavones consumed in aglycone form reduced depressive symptoms as assessed by the Hospital Anxiety and Depression Scale and also reduced anxiety as assessed by the Athens Insomnia Scale [331]. In contrast to the benefit of this dose, this eight-week trial found that a very low dose of isoflavones (12.5 mg/day) lacked efficacy. Finally, Estrella et al. (2014) found that over a three-month period 100 mg/day isoflavones reduced depressive symptoms in clinically depressed postmenopausal women to a similar extent as Zoloft (50 mg/day) and Prozac (10 mg/day) [332]. In addition, the combination of Zoloft and isoflavones resulted in a greater reduction in symptoms than the other three individual treatments.

## 13. Skin Health

The impact of soy and more specifically isoflavones, on skin health including wrinkles, is another area for which encouraging preliminary data exist. Interest in the effects of isoflavones on overall skin health is not surprising given that they bind to ERs, which are present in the skin [333,334]—and that estrogen therapy is thought to improve a number of skin parameters [335,336,337,338] including skin elasticity [339], water-holding capacity [340], pigmentation [341] and vascularity [342]. Skin appendages, such as hair follicles, are also influenced by estrogens [343].

Several trials suggest that isoflavones help to reduce wrinkles but all of them have design limitations. In one study, which lacked a true control group, two groups of 20 healthy postmenopausal women aged 50 to 65 years were instructed to consume their usual diet with or without 20 g/day of an isoflavone-rich soy protein for three months [344]. There were statistically significant improvements in facial-skin wrinkling, discoloration and overall appearance in the supplemented group. In another study, which involved only 26 premenopausal Japanese women, over a 3-month period, use of supplements that provided 40 mg/day isoflavones led to a statistically significant decrease in fine wrinkles, whereas no change occurred in the placebo group [345]. Finally, a 14-week trial conducted by Jenkins et al. (2013) involving 159 postmenopausal women found that a beverage containing isoflavones (plus other bioactives) statistically significantly reduced wrinkles by on average 10%; there was also a positive correlation between baseline wrinkles and the response to the isoflavone-containing beverage; that is, the greater the wrinkle depth at baseline, the greater the improvement [346]. There was also a statistically significant increase in collagen synthesis.

## 14. Developmental Effects

Establishing healthy eating habits early in life is important because childhood eating habits track into adulthood, and changing adult dietary behavior is difficult [347,348,349,350,351]. Also, evidence suggests that healthy behaviors during childhood and adolescence can affect the risk of developing certain chronic diseases later in life [352,353,354,355]. Not surprisingly, compared to adults, relatively little soy research has involved children. Nonetheless, research has shown that as in adults, soy protein lowers LDL-cholesterol [356,357,358,359,360]. As discussed previously, evidence also suggests that eating soy early in life reduces risk of breast cancer [222,223,224,225,226,227,228].

The classification of isoflavones as phytoestrogens has led to investigation of the possible hormonal effects of soy consumption in children. Although only very limited clinical research addressing this issue has been conducted the evidence suggests neither soy nor isoflavone intake affects endogenous hormone levels [361,362,363].

There is also interest in understanding the impact of diet on pubertal development because pubertal characteristics are occurring at an earlier age [364,365]. Many factors likely contribute to this trend such as increasing adiposity. Epidemiologic studies have found that both total and animal protein intake is associated with earlier menarche and the development of early pubertal characteristics [366,367].

Two small Korean epidemiologic studies noted that urinary isoflavones in children with precocious puberty were higher than in children serving as controls [368,369]. However, a US cross-sectional study involving Seventh-day Adventist (SDA) girls (*n* = 327; age range 12 to 18 years; mean age, 15 years) found that soy intake was unrelated to the age of onset of menses [370]. The mean number of servings of soyfoods among the adolescent girls was 12.9 per week and 21.1% of the girls consumed soyfoods ≥4 times/week [370].

## 15. Fertility/Reproduction

There are number of issues involving soyfoods that have given rise to quite lively discussions in the peer-reviewed literature. One of those somewhat ironically, given the large populations of Asian countries that have historically consumed soy, is the possible adverse impact of soy on fertility On the other hand, breeding problems have been noted in some animal species in response to isoflavone ingestion [371,372,373,374] although it was subsequently shown that these issues arose because of either excessive intake [375] or because of differences in isoflavone metabolism between animals and humans [69,81,376,377].

In women, soyfoods appear to increase the length of the menstrual cycle although ovulation is not prevented it is simply delayed by one day [254]. According to limited epidemiologic data, this minor effect on menstrual cycle length could help to decrease breast cancer risk [378]. There is actually some evidence that isoflavones aid fertility. A prospective study found that among 315 women who collectively underwent 520 assisted reproductive technology cycles soy isoflavone intake was positively related to live birth rates [379]. Similarly, among women undergoing in vitro fertilization, soy consumption appeared to negate the adverse reproductive effects of the endocrine disruptor bisphenol A (BPA) [380]. Although the low isoflavone intake among the soy-consumers (mean intake, 3.4 mg/day) in this study raises doubt about the plausibility of these findings, they do agree with animal data [381,382].

In men, a small pilot cross-sectional study found that very modest soy consumption was associated with lower sperm concentration (sperm count was not decreased) but there were many weaknesses to this study [383]. In fact, much of the decreased sperm concentration occurred because there was an increase in ejaculate volume in men consuming higher amounts of soy, a finding which seems biologically implausible. Furthermore, this same research group subsequently found in a cross-sectional study involving 184 men from couples undergoing in vitro fertilization that male partner's intake of soyfoods and soy isoflavones was unrelated to fertilization rates, proportions of poor quality embryos, accelerated or slow embryo cleavage rate, and implantation, clinical pregnancy and live births [384].

Finally, and most importantly, all three of the clinical studies conducted show that isoflavones have no effect on sperm concentration or quality [385,386,387]. Interestingly, a case report indicated that daily isoflavone supplementation for six months in the male partner of an infertile couple with initially low sperm count led to normalization of sperm quality and quantity [388].

## 16. Male Feminization

Two case reports describing feminizing effects that allegedly occurred as a result of soyfood consumption have been published [389,390]. However, in both cases the individuals were said to have consumed 360 mg/day isoflavones (~9-fold greater than the mean intake among older Japanese men) in the context of unbalanced and likely nutrient-deficient diets since soyfoods accounted for the vast majority of calories consumed. Furthermore, in contrast to the rise in circulating estrogen levels noted in one case [389], no effects on estrogen levels have been noted in numerous clinical studies in which men were exposed to as much as 150 mg/day isoflavones [391].

Similarly, the drop in testosterone levels noted in the other case [390] is as already noted, inconsistent with the preponderance of the clinical data showing neither soy nor isoflavone supplements affect testosterone levels [283]. More specifically, a systematic review and meta-analysis that included 15 placebo-controlled treatment groups with baseline and ending measures and an additional 32 reports involving 36 treatment groups found no effects of soy protein or isoflavone intake on testosterone, sex hormone binding globulin, free testosterone or the free androgen index [283]. Studies published subsequent to this meta-analysis have reached similar conclusions [168,392,393,394]. The two aforementioned case reports simply illustrate that consuming excessive amounts of essentially any food can potentially lead to abnormalities [389,390].

## 17. Thyroid Function

Concerns about the anti-thyroid effects of soy are based primarily on in vitro research [395,396] and studies in rodents administered isolated isoflavones [397,398]. Although several cases of goiter were attributed to the use of soy infant formula, this problem was eliminated in the mid-1960s with the advent of iodine fortification of the formula [399,400,401]. A comprehensive review published in 2006 that included 14 clinical trials found that the totality of the evidence showed that neither soyfoods nor isoflavones adversely affect thyroid function in euthyroid men or women [402]. Studies published since this review [403,404,405,406,407], which include two that were three years in duration [408,409], are supportive of this conclusion as is the conclusion of the EFSA that isoflavone supplements do not affect thyroid function in postmenopausal women [108].

Soyfoods may increase the amount of thyroid medication needed by hypothyroid patients, not because of an effect on the thyroid, but because soy protein may interfere to some extent with the absorption of levothyroxine [410,411,412,413]. Soy is not unique in this regard however as many herbs and drugs and fiber and calcium supplements have similar effects [414,415,416,417,418,419,420,421,422]. In any event, it is not necessary for thyroid patients (with the exception of infants with congenital hypothyroidism) to avoid soyfoods since thyroid medication is taken on an empty stomach and dosages can easily be adjusted to compensate for any effects of soy [423].

Finally, there is concern that soy may worsen thyroid function in those whose thyroid function is compromised such as subclinical hypothyroid patients and those whose iodine intake is marginal. The concern about the latter is based on the potential for isoflavones rather than the amino acid tyrosine to be iodinated, thereby inhibiting the synthesis of thyroid hormone [424]. However, clinical research published in 2012 indicates that the iodination of isoflavones is negligible and clinically irrelevant [425]. One small British study did find that modest isoflavone exposure (16 mg/day) increased the likelihood of progressing from subclinical to overt hypothyroidism [166]. These results are surprising because the progression of subclinical to overt hypothyroidism among Japanese patients is not elevated [426] nor are rates of hypothyroidism elevated in Japan [427]. It is notable that in the British study isoflavone exposure caused marked and statistically significant reductions in systolic and diastolic blood pressure, insulin resistance and CRP.

## 18. Effects on Endometrial Tissue

Endometrial cancer (cancer of the corpus uteri) represents the most common gynecological malignancy in the industrialized world and is the seventh most common cancer among females although incidence and mortality rates vary markedly among geographical regions and countries [428]. The highest rates are in the United States and Europe and the lowest in Asia and Africa [429]. There is concern that because of the presence of isoflavones soyfoods could increase risk of developing endometrial cancer and stimulate the growth of existing endometrial tumors. Ever users of unopposed estrogen therapy are about two to three times more likely to develop endometrial cancer as never users [430,431,432].

However, after reviewing 25 clinical studies that measured endometrial thickness and nine that measured histopathological changes the EFSA concluded that isoflavones do not adversely affect the endometrium [108]. Interestingly, a recently published meta-analysis of the clinical data found that when all studies (*n* = 23; 2167 participants) were included in the analysis there was no effect of isoflavones on endometrial thickness overall whereas there was a significant (*p* = 0.04) decrease in thickness when considering only the seven North American trials which involved 726 women [433]. On the other hand, there was small increase in thickness among women involved in the three Asian trials but none of these studies actually intervened with soybean-derived isoflavones.

Furthermore, a recent meta-analysis of 10 observational studies (8 case-control, 2 prospective) found soy intake was inversely associated with endometrial cancer risk with an overall risk estimate (RE) of 0.81 (95% CI: 0.72, 0.91) [434]. Subgroup analyses found statistically significant protective effects for both Asian (RE: 0.79, 95% CI: 0.66, 0.95) and non-Asian (RE: 0.83, 95% CI: 0.71, 0.96) populations. Finally, Bitto et al. (2010) found that in a group of premenopausal women with non-atypical endometrial hyperplasia, genistein (54 mg/day) improved symptoms after six months to approximately the same degree as norethisterone [435]. Hence, Bitto et al. (2010) concluded that genistein might be useful for the management of endometrial hyperplasia without atypia in women that cannot be treated with progestin [435].

## 19. Conclusions

Soyfoods have become increasingly popular in non-Asian countries. Their versatility allows them to easily be incorporated into Western diets and therefore provides a convenient way to exploit the nutritional advantages of legumes, which often play an underutilized role in North America and many European countries. However, the macronutrient composition of the soybean is different from other legumes. Also, soy protein is higher in quality than other legume proteins and the soybean is a good source of both essential fatty acids. Soy protein also directly lowers circulating LDL-cholesterol levels and may also modestly lower blood pressure. Replacement of commonly-consumed sources of protein in Western diets by soyfoods may also lead to a favorable change in the fatty acid content of the diet.

The most distinctive aspect of the soybean is its high isoflavone content. Isoflavones are proposed as having a number of health benefits although not surprisingly, the degree to which the evidence supports these claims varies. For example, there is solid evidence in support of isoflavones alleviating hot flashes and improving arterial health in menopausal women whereas the evidence that they reduce risk of breast and prostate cancer, not surprisingly, is more preliminary. Concerns that the estrogen-like properties of isoflavones produce untoward effects in some subpopulations, such as postmenopausal women, are not supported by the clinical and epidemiologic research. Evidence indicates soyfoods can be safely consumed by all individuals except those who are allergic to soy protein, which is relatively uncommon in comparison to the number of individuals allergic to many other commonly-consumed foods [436,437,438].

When adding soy to the diet it is important to consider the overall nutritional quality of a particular soyfood since many Westernized soyfoods include a variety of non-soy ingredients. There are no formal recommendations for soy intake beyond the 25 g/day soy protein established by the US FDA as the threshold intake for cholesterol reduction. However, population and clinical studies involving adults suggest benefits are associated with approximately two to four servings per day. Ideally, soyfoods are incorporated into the diet by displacing less healthy foods and as part of an overall healthy diet designed to lower risk of chronic disease such as the approach represented by the portfolio diet [439].

## Figures and Tables

**Table 1:** Nutrient composition of boiled soybeans and selected legumes per cup (source, reference [11]).

Legume/USDA Nutrient Database Number	kcal	Macronutrients % kcal/g per Cup			Fiber (g)	Minerals (mg) per Cup						Vitamins (Amounts per Cup) ^1^						
		Protein	Fat	CHO ^2^		Ca	Iron	Zinc	P	K	Mg	DFE ^3^	B2	B6	B3	Thiamin	α-Toc ^4^	K
Soybeans/16109	296	42.3/31.3	46.9/15.4	19.4/14.4	10.3	175	8.84	1.98	421	886	148	93	0.490	0.402	0.686	0.267	0.60	33.0
Lentils/16070	230	31.1/17.9	2.9/0.8	69.3/39.9	15.6	38	6.59	2.51	356	731	71	358	0.145	0.352	2.099	0.335	0.22	3.4
Pinto beans/16043	245	25.2/15.4	4.1/1.1	73.2/44.8	15.4	79	3.57	1.68	251	746	86	294	0.106	0.392	0.544	0.330	1.61	6.0
Navy beans/16038	255	23.5/15.0	4.0/1.1	74.4/47.4	19.1	126	4.30	1.87	262	708	96	255	0.120	0.251	1.181	0.431	0.02	1.1
Chickpeas/16057	269	21.6/14.5	14.2/4.3	66.9/45.0	12.5	80	4.74	2.51	276	477	79	282	0.103	0.228	0.863	0.190	0.57	6.6
Kidney beans/16028	225	27.3/15.4	3.5/0.9	71.8/40.4	11.3	62	3.93	1.77	244	717	74	230	0.103	0.212	1.023	0.283	0.05	14.9
Black beans/16015	227	26.9/15.2	3.7/0.9	71.9/40.8	15.0	46	3.61	1.93	241	611	120	256	0.101	0.119	0.869	0.420	1.50	5.7
Adzuki beans/16002	294	23.5/17.3	0.7/0.2	77.5/57.0	16.8	64	4.60	4.07	386	1224	120	278	0.147	0.221	1.649	0.264	NR	NR
Great Northern/16025	209	28.2/14.7	3.4/0.8	71.4/37.3	12.4	120	3.77	1.56	292	692	88	181	0.104	0.207	1.205	0.280	NR	NR
Lima beans/16372	216	27.1/14.7	3.0/0.7	72.7/39.3	13.2	32	4.49	1.79	209	955	81	156	0.103	0.303	0.791	0.303	NR	3.8

^1^ Units are mg for vitamins B2, B6, B3, thiamin and vitamin E; µg for folate and vitamin K; ^2^ CHO, carbohydrate; ^3^ DFE, dietary folate equivalents; ^4^ α-Toc, α-tocopherol.

**Table 2:** Fatty acid composition of soybean oil by seed coat color (grams per 100 g of oil).

Type of Fat	Soybeans ^1^	Black (*n* = 5) ^2^		Brown (*n* = 4) ^2^		Green (*n* = 2) ^2^		Yellow (*n* = 7) ^2^	
		Average	Range	Average	Range	Average	Range	Average	Range
Total saturated	15.6	14.98	14.67–15.18	10.28	10.10–10.49	13.76	13.03–13.76	11.76	7.08–22.68
Palmitic (% of saturated)	73.4	11.39	11.21–11.64	6.06	5.86–6.19	10.28	9.58–10.97	7.76	3.54–10.61
Stearic (% of saturated)	24.7	3.59	3.03–4.05	4.22	4.11–4.36	3.48	3.45–3.51	4.00	3.48–6.08
Monounsaturated (oleic)	23.8	19.65	17.13–22.99	41.15	41.01–41.48	24.80	23.51–26.08	29.35	21.71–36.03
Total polyunsaturated	60.7	62.71	58.66–66.03	46.11	45.93–46.47	58.75	56.83–60.96	55.55	44.95–61.84
Linoleic (% of PUFA ^3^)	88.2	53.12	50.63–55.14	42.88	42.63–43.13	50.71	48.88–52.53	50.18	48.31–53.99
α-Linolenic (% of PUFA)	11.8	9.59	7.99–10.78	3.23	3.10–3.31	8.05	7.76–8.33	5.37	3.37–9.07

^1^ Source, reference [11]; ^2^ Source: reference [44]; ^3^ PUFA, Polyunsaturated fatty acids.

**Table 3:** Meta-analysis of clinical studies evaluating the effects of soy protein on lipid levels.

Author/(Reference)	Year Published	Number of Trials	*n* ^1^	Change from Baseline		
				LDL-C ^2^	HDL-C ^3^	Triglyceride
Tokede [126]	2015	22 ^4^		−5.88 mg/dL	+1.42 mg/dL	−7.12 mg/dL
		13 ^5^		−7.62 mg/dL	+1.15 mg/dL	−7.48 mg/dL
Benkhedda [118]	2014	59	3731	−4.0%	+0.03 mmol/l	−0.06 mmol/L
Yang [127]	2011	7 ^6^	158	−0.30 mmol/L	+0.05 mmol/l	−0.22 mmol/L
Anderson [125]	2011	20 ^7^	1946	−5.5%	+3.2%	−10.7%
Jenkins [122]	2010	22	757	−4.2%	NR ^8^	NR ^8^
Harland [124]	2008	10	2913	−6.0%	+4.6%	−5.3%
Hooper [128]	2008	39	2747	−0.19 mmol/L	0.02 mmol/L	NR ^8^
Reynolds [129]	2006	36	1387	−4.0%	+0.77 mg/dl	−6.26 mg/dL
Zhan [123]	2005	33	1749	−5.3%	+3.0%	−7.3%
Weggemans [130]	2003	21	959	−4.0%	+3.0%	NR ^8^

^1^ Refers to participants for LDL-cholesterol measurements; ^2^ LDL-C, LDL-cholesterol; ^3^ HDL-C, high-density-lipoprotein cholesterol; ^4^ Trials intervening with soy protein containing isoflavones; ^5^ Trials intervening with soy protein without isoflavones; ^6^ All participants had type 2 diabetes; ^7^ Refers to parallel studies only; ^8^ NR, not reported.

**Table 4:** Experimental details of prospective epidemiologic studies included evaluating the impact of post-diagnosis soy intake on breast cancer recurrence and survival ^1^.

Author/(Reference)	Location	*n*	Follow up (Year)	Deaths	Recurrences
Zhang [259]	China	616	4.3	79	NA ^2^
Caan [256]	United States	3088	7.3	271	448
Kang [258]	China	524	5.1	154	185
Shu [257]	China	5042	3.9	444	534
Guha [255]	United States	1954	6.3	NA ^2^	282
Totals	---	11,224	---	948	1,449

^1^ Adapted from reference [260]; ^2^ NA, not applicable.

**Table 5:** Effects of isoflavones on the decrease in hot flash frequency according to the genistein content of the supplement ^1^.

Genistein Dose (mg/Day)	Number of Trials	*n*	Fixed Effects Model			Random Effects Model		
			Percent	95% CI	*p* Value	Percent	95% CI	*p* Value
≤18.8	7	596	−12.47	−17.34, −7.60	<0.00001	Same as Fixed Effects Model		
>18.8	6	600	−26.50	−33.11, −19.90	<0.00001	−29.13	−43.09, −15.17	<0.0001
Test for Subgroup Difference			*p* = 0.0008			*p* = 0.03		

^1^ Source: reference [305].
